# A Subtraction Genomics-Based Approach to Identify and Characterize New Drug Targets in *Bordetella pertussis*: Whooping Cough

**DOI:** 10.3390/vaccines10111915

**Published:** 2022-11-12

**Authors:** Alam Jamal, Sadaf Jahan, Hani Choudhry, Irfan A. Rather, Mohammad Imran Khan

**Affiliations:** 1 Department of Biochemistry, Faculty of Science, King Abdulaziz University, Jeddah 21589, Saudi Arabia; 2 Department of Medical Laboratory Sciences, College of Applied Medical Sciences, Majmaah University, Al-Majmaah 15362, Saudi Arabia; 3 Centre of Artificial Intelligence for Precision Medicines, King Abdulaziz University, Jeddah 21589, Saudi Arabia; 4 Department of Biological Sciences, Faculty of Science, King Abdulaziz University, Jeddah 21589, Saudi Arabia

**Keywords:** *Bordetella pertussis*, whooping cough, vaccination, subtractive genomics, proteins

## Abstract

*Bordetella pertussis* is a Gram-negative bacterium known to cause pertussis or whooping cough. The disease affects the respiratory system and is contagious. Pertussis causes high mortality among infants aged less than one-year-old, although it can affect anyone of any age. Globally, 16 million cases of pertussis were reported in 2008, 95% of which were in developing nations, and approximately 195,000 children died from the disease. Under a computational subtractive genomics approach, the total proteome of a pathogen is gently trimmed down to a few potential drug targets. First, from NCBI, we obtained the pathogen proteins followed by CD hit for removal of duplicate proteins. The BLAST step was applied to find non-similar proteins, and then, we applied BLAST to these non-similar bacterial proteins with DEG to find essential bacterial proteins. After this, to find the location, these vital proteins were screened via PSORTb; the majority of proteins were in cytoplasm. The KASS server was used to determine the involvement of these proteins in the metabolic pathways of bacteria, and KEGG was applied to find the unique metabolic pathways of the pathogen. Finally, we applied BLAST to these vital, unique, and non-similar proteins with FDA-approved drug targets, and four proteins of the *B. pertussis* strain B1917 were identified that might be powerful drug targets. A variety of therapeutic molecules could be designed to target these proteins in order to treat infections caused by bacteria.

## 1. Introduction

Pertussis is known for intense, unmanageable coughing, causing breathing difficulties. After coughing fits, patients with pertussis are mainly required to take deep breaths, which results in a “whooping” sound. Vaccination is a fruitful approach for preventing and controlling pertussis; however, the incidence of pertussis persists in many countries. Current epidemiological and clinical studies have revealed the revival of pertussis. Pertussis is a notable cause of infant death. Pertussis remains primarily a childhood infection, although it is spreading to adults and teenagers [1].

According to WHO evaluations, 16 million pertussis cases were reported globally in 2008, 95% of which were in developing nations, and approximately 195,000 children died from this illness. Yeung et al. used a model based on facts presented by the World Health Organization (WHO) in 2014 to evaluate 24.1 million cases and deaths, 160,700 of which were in children under the age of five [2].

Although the introduction of the vaccine has dramatically reduced disease load, a recurrence of infection has been observed among highly vaccinated populations in the 1990s [3]. Countries including the United Kingdom, the Netherlands, the United States, and Australia have seen incredibly high infant mortality rates from 2010 to 2012 [4]. Several factors contribute to bacterial resistance, including adaptation to vaccines and reduced immunity induced by vaccines.

Macrolides are often used to treat pertussis, but in China and Iran, macrolide-resistant *B. pertussis* (MRBP) strains have been observed [5]. Since the early 2010s, MRBP has been secluded with increasing frequency (57.5–91.9%) in China [6]. Surprisingly, the prevalence of pertussis has risen continuously for the past 20 years in nations with high vaccination rate.

In China, *B. pertussis*, which shows resistance to erythromycin (EM), is another trouble in many cases, as EM is the primary treatment for disease [7]. In the subsequent years, high numbers of EM-resistant isolates have been identified in many megacities: 57.4% in Shanghai [8], 2016–2017; 87.5% in Xi’an [8], 2012–2013, and even 91.4% in Beijing [9], 2013–2014.

Since the 1980s, erythromycin has been used to treat pertussis in China, with MRBP being used in small numbers before 2008. Although, since that time, a sharp macrolide resistance increase has been noticed in northwest, east, and north China, where above fifty percent of B. pertussis isolates were discovered, showing resistance to macrolides. Therefore, all over China, MRBP is predicted to have prevailed. In bacteria for erythromycin resistance, there are two known mechanisms: erythromycin-resistant methylase (erm) genes procurement [10] and the 23S rRNA (rrn) gene mutations causing modifications in the structure that avert the erythromycin attachment [11]. Almost all reports have validated that MRBP resistance is arbitrated due to the latter mechanism and mutation that concentrates on the transition of A2047G. To treat the MRBP disease, high level of erythromycin is not viable. The resistance arbitrated by this mutation is also associated with azithromycin. For MRBP treatment, azithromycin is also no longer helpful. It is likely that the MRBP ratio is bigger in other states but is being ignored because the diagnostic methods are insufficient [12].

The panic raising of strains that show drug resistance is important in finding a potent therapy as the current drugs are ineffective in various infections caused by bacteria [13]. The novel used technique to beat the resistive pathogens to detect the specific and novel targets for the drug from the complete bacterial proteins. Different approaches have been used that concentrate on finding unknown drug target sites in the literature [14,15,16]. Subtractive proteomic analysis is the most reasonable approach to detecting new targets for the drug within these circumstances [16,17]. To detect some novel drug targets against *B. pertussis* strain B1917, in this work, we applied a computational subtractive genomics analysis method. First, we retrieved the complete proteome of our target bacteria from NCBI. These proteins were then subjected to CD Hit to leave only non-duplicate and more than 100 amino acid proteins, because there are less chances that those proteins which have less than 100 amino acids are essential for pathogen. After this, the BLAST step was applied to find non-similar proteins, and then we applied BLAST to these non-similar bacterial proteins with DEG for finding of essential bacterial proteins. Then, PSORTb was used to find location of proteins; most of proteins were in Cytoplasm. Then, KASS server was used to find the involvement of these proteins in metabolic pathways of bacteria, and KEGG was applied for finding unique metabolic pathways of pathogen. Finally, we applied BLAST to these vital, unique and non-similar proteins with FDA-approved drug targets. Then, these short-listed protein sequences were further compared with human gut microbiome, because many microbes in human gut carry out very important functions for their host and live symbiotically there. Therefore, we selected only those proteins which were not present in gut microbiomes to avoid side effects.

## 2. Materials and Methods

**Figure 1:**
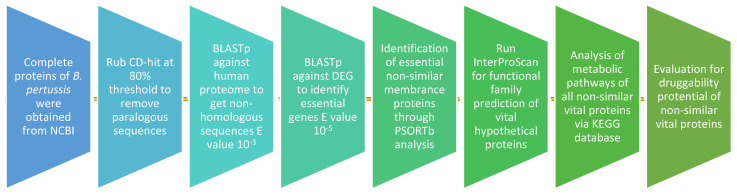
Flow chart of the study design.

For the recognition of vital proteins in the *B. pertussis* strain B1917, Subtractive Genomics method was applied, which was then examined to identify the expected drug targets. To estimate their draggability capacity, the recognized drug targets were passed via a database of Drug Bank. An overview of the overall workflow is shown in Figure 1.

### 2.1. Complete Bacterial Proteome Retrieve

The total proteome of *B. pertussis* strain B1917 was retrieved from NCBI in FASTA format and the sequences were processed in the same format [18].

### 2.2. Finding Duplicate Proteins

CD-HIT was run to recognize duplicate protein sequences with a recognition threshold of 0.8 (i.e., 80%). The CD-HIT suite is frequently used to compare and group proteins that meet the sequence identity criteria. It produces a list of clusters as well as one representative sequence for each cluster. From the total proteome of *B. pertussis* strain B1917, duplicate sequences and proteins which have less than 100 amino acids were separated, and in further steps we used only non-duplicate and proteins which have more than 100 amino acids [19].

### 2.3. Detection of Non-Similar Proteins

To detect proteins in *B. pertussis* that differ from proteins in humans, BLASTp was used at E value 10^−3^ of the threshold. The resulting proteins included non-homologous and homologous successions. Protein successions that were homologous sufficiently with the human were removed and non-similar sequences were selected for further examination [20].

### 2.4. Detection of Vital Proteins in B. pertussis

Using a DEG database consisting of the bacteria’s vital proteins with a threshold of 10^−5^ E value, the non-similar successions were blast (BLASTp) with DEG. The successions of proteins with high similarity to the database of essential genes were selected as protein sequences that are vital for bacteria continuation. Considering these essential proteins as vital for the survival of pathogen, the sequences were selected for further analysis [21].

### 2.5. The KEGG Metabolic Pathways Investigation

The KAAS server was applied to predict sequences of proteins that participate in different bacterial metabolic pathways. In addition, KEGG was used to find unique metabolic pathways of target bacteria [22]. Only unique metabolic pathways were selected by comparing the host and bacterial metabolic pathways in KEGG. The unique pathways are only present in pathogens, not in the host, so by targeting the proteins which are involved in these unique metabolic pathways, we face fewer chances of side effects on host pathways.

### 2.6. Subcellular Location Foretelling

To predict the subcellular localization of non-homologous and essential proteins, version 3.0 of PSORTb was applied. The primary basis was to run a Subcellular localization BLAST that uses non-homologous and necessary protein sequences; BLASTp runs against the proteins database of known subcellular localization. PSORTb provides divine results for different subcellular localization of proteins, which involve cytoplasmic membrane, wall of the cell, unknown, extracellular, and cytoplasm [23].

### 2.7. Hypothetical Proteins Family Prediction

We applied InterProScan to predict the functional family classification of hypothetical sequences whose functions are unknown, but their sequences are known. The InterProScan predicts protein family prediction on the basis of functional group. The proteins sequences were uploaded to InterProScan in FASTA format.

### 2.8. Chosen Sequences Draggability Potential

Via BLASTp, similar, vital, and hypothetical protein sequence screenings were evaluated compared with the Drug Bank database that included a number of protein targets with respect to FDA-approved drug IDs. BLASTp search against the database of Drug Bank with Default parameter of E value 10^−3^ was applied to detect new drug targets. A list of proteins with a high degree of similarity to FDA-approved drug targets were compiled [24].

### 2.9. Human Gut-Metagenomes Screening

A wide range of organisms contribute to a healthy body, and this is a mutually beneficial relationship. These microorganisms play important roles in the human body such as immune system modulation, hormone production, inhibiting the growth of harmful species, and vitamin synthesis. Inhibiting these proteins will therefore be detrimental to the host. Therefore, to remove these similar proteins, the targeted proteins of B. pertussis were compared to the protein sequences of the gut flora at the Human Microbiome Project database server by BLASTp with an E-value cutoff score of 1 [25].

## 3. Results and Discussion

The principal objective of this study was to identify a potent new drug target against *B. pertussis*, a pathogen characterized by rapid antibiotic resistance such as erythromycin (EM), and Macrolides in some regions. Despite the development of antibiotic resistance, it is alarming that previously discovered antibiotics are becoming less effective. Consequently, subtractive genomics is an excellent approach for finding novel drug targets in bacteria. In order to identify drug targets that meet the potentiality criteria, it is necessary to identify targets that are non-homologous to the host, vital to pathogens, and involve the essential metabolic processes involved in pathogens. Figure 1 shows the flow chart of the study design, and the different outcomes of each step are shown in Table 1. The total proteome of *B. pertussis* strain B1917 consists of 3550 protein sequences obtained from NCBI. The 3550 protein sequences were then subjected to CD-HIT with a threshold of 0.8, removing paralogues and less than 100 amino acid protein sequences. The step identified 431 duplicates, and 204 less than 100 amino acid sequences were removed, leaving 3085 proteins. Then, 3085 non-duplicate proteins were subjected to BLASTp against the genome of humans. This outcome displayed 2025 sequences that were non-similar to the host’s genome. When used as targets for the drug in the patients, proteins similar to the human genome may cause unpleasant side effects and toxic reactions. Then, the proteins of *B. pertussis* were passed *via* BLASTp against DEG with a 10^−5^ E-value threshold to obtain a set of vital 708 proteins. The genes involved in these genes are crucial for the survival of the pathogen, so targeting these genes may reduce the risk of bacterial infections. These genes should be different from the host’s genome; the clench is a considerable pledge to be used in targets for drugs in specific species. In our study, we selected only essential proteins that are involved in unique metabolic pathways of *B. pertussis* and show significant differences from FDA-approved drug targets that are not found in human gut bacteria.

### 3.1. Subcellular Location of Essential and Non-Hologous Proteins

**Figure 2:**
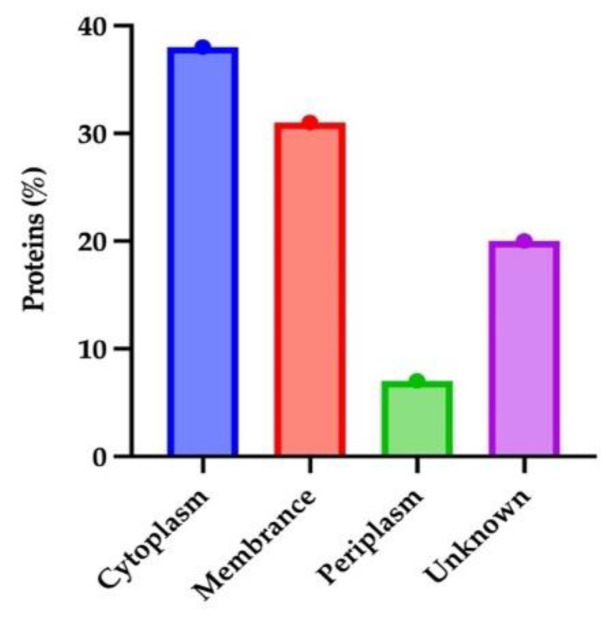
Subcellular localization of vital non-homologous proteins of *B. pertussis*.

Protein location is an important requirement for determining its activity. Therefore, proteins need to be located in a specific location to perform their function. Investigating the proteins can provide insight into protein subcellular localization. PSORTb is the most accurate approach for predicting subcellular locations. The vital and non-similar sequences were introduced to PSORTb, resulting in most of the protein sequences (38%) in the cytoplasm region (Figure 2). The 2nd highest number of proteins was present in the cytoplasm membrane. Because of their prominent positions in the outer membrane, these proteins are frequently the focus of antibody-based treatments and vaccinations. Outer Membrane Proteins are engaged in crucial processes such as adhesion, biofilm formation, regulation of quorum sensing, or the extrusion of hazardous chemicals; their surface-exposed epitopes make them accessible for antibody or T-cell receptor recognition. Because these proteins are often highly produced and conserved, there are often more of them to use as antigens [26]. Antigens chosen cause strong immunological and memory responses, providing protection that is both targeted and long-lasting, suggesting that proteins of the cytoplasm membrane may be the dynamic vaccine targets [27].

### 3.2. Vital, Non-Homologous and Hypothetical Proteins Family Predection

Hypothetical proteins have known sequences but unknown functions. There are a number of sequences that are hypothetical in bacteria that causes infection. The excellent approach for predicting the function of hypothetical sequences is to classify them based on homogeneity in functional group sequence. One of the best approaches to categorizing sequences in functional groups/families is InterProScan. In *B. pertussis*, 19 hypothetical protein sequences were present. For this foretelling, we used the InterProScan method. Prohibitin, Stomatin/HflK/HflC family, Maleate isomerase, and Arylmalonate decarboxylase were the most common families.

### 3.3. KEGG Metabolic Pathways Analysis

The vital non-homologous proteins were introduced into the KASS server. The server was used to detect the contribution of the submitted protein sequences in different vital metabolic pathways active in the pathogen. A total of 708 sequences of proteins were subjected to this server. The different pathways of metabolism in which the *B. pertussis* participate are glycolysis/gluconeogenesis, aldarate and ascorbate metabolism, pentose phosphate pathway, nucleotide sugar metabolism, pyruvate metabolism, propanoate metabolism, C5-branched dibasic acid metabolism, carbon fixation in photosynthetic organisms, butanoate metabolism, metabolism of methane, two-component system, folate biosynthesis, nucleotide excision repair, amino sugar metabolism, dicarboxylate metabolism, pyrimidine metabolism, oxidative phosphorylation, fatty acid biosynthesis, glyoxylate metabolism, sulfur metabolism, and metabolism of glycine.

**Figure 3:**
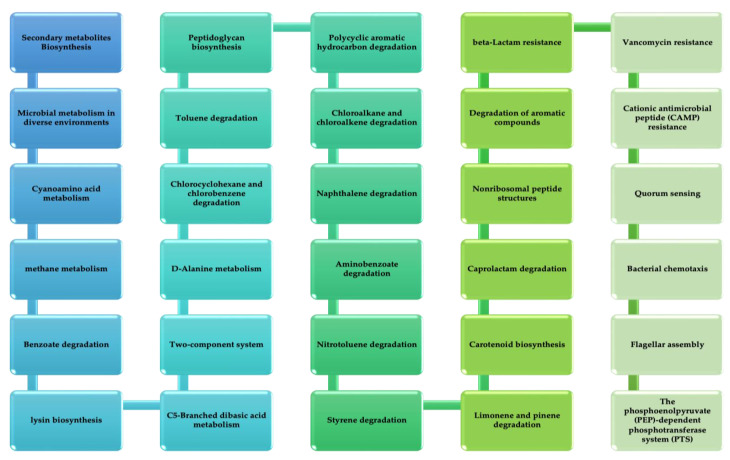
Unique metabolic pathways of *B. pertussis* from KEGG.

Pathways of carbon fixation are found in prokaryotes, methionine and cysteine metabolism, metabolism of nitrogen, ketone bodies degradation and synthesis, metabolism of glycerophospholipid, metabolism of purine, leucine, valine and isoleucine biosynthesis, metabolism of aspartate, mismatch repair, alanine and glutamate, proline and arginine metabolism, lysine biosynthesis, serine and threonine metabolism, hypotaurine and taurine metabolism, breakdown of fatty acid, histidine metabolism, arginine biosynthesis, riboflavin metabolism, biosynthesis of monobactam, bacterial secretion system, biosynthesis of CoA and pantothenate, breakdown of leucine, valine, and isoleucine, metabolism of D-ornithine and D-arginine, metabolism of chlorophyll and porphyrin, degradation of lysine, biotin metabolism metabolism of glycerolipid, terpenoid backbone biosynthesis, biofilm formation-pseudomonas aeruginosa, cationic antimicrobial peptide (CAMP) resistance, phenylalanine metabolism, biosynthesis of phenylalanine, biosynthesis of tryptophan and tyrosine, beta-alanine metabolism, seleno compound metabolism, biosynthesis of lipopolysaccharide, biosynthesis of peptidoglycan, nicotinate and nicotinamide metabolism, ribosome, protein export, DNA replication, homologous recombination, pertussis, ABC transporters, quorum sensing, thermogenesis, bacterial chemotaxis, flagellar assembly, PPAR signaling pathway, vancomycin resistance and beta-lactam resistance. Figure 3 shows distinctive pathways of metabolism. The proteins related to these pathways are the most potent drug targets; due to the non-availability of antagonist pathways in humans, the side effects are sporadic.

### 3.4. Shortlisted Sequences Drug Ability Capacity

This study was further augmented by screening out the draggability capacity of all the shortlisted vital proteins. In the database of Drug Bank, it results from the recognition of eight proteins that resemblance the FDA-sanctioned drug targets. Information on chosen proteins with IDs of the target is shown in Table 2. The eight targets of drugs resembling Drug Bank homologs are correlated to neuraminidase, src-p60 phosphoprotein, MHC HLA-DC3-beta, Ig heavy chain V-A2 region BS-1, and MHC H2-IA-alpha. All the eight proteins can be dynamic drug targets, since, in Drug Bank, all have one analog with at least 30% homogeneity in sequence.

### 3.5. Human Gut-Metagenomes Screening

To target only the proteins present in the *B. pertussis*, we perform blastp against the human microbiome database, which results in only four protein sequences that have no similarity to the human microbiome database. These four proteins are present in cytoplasm membrane and can be a valuable vaccine target as shown in Table 3.

**Figure 4:**
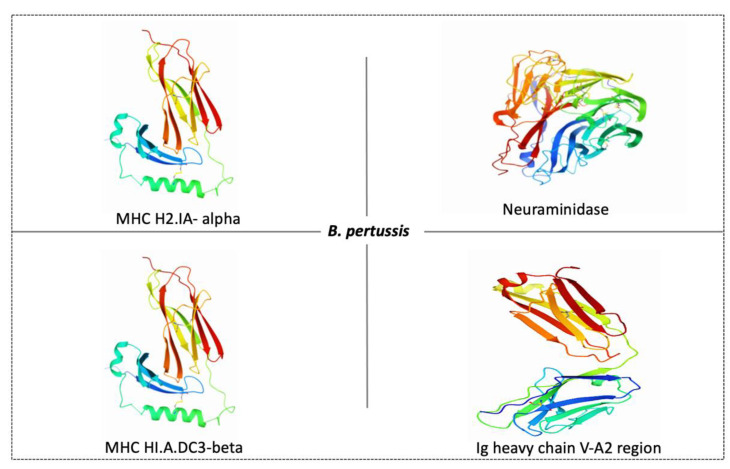
Possible protein targets and their structures for vaccine development against B. pertussis. PDB ID of MHC H2-IA-alpha is 1MHC, neuraminidase is 5TSP, *MHC HLA-DC3-beta is 3HPJ* and Ig heavy chain V-A2 region is 1IGM.

The function of neuraminidase is contributing to the formation of biofilm while MHC H2-IA-alpha and *MHC HLA-DC3-beta* enables MHC class II protein complex binding; the function of Ig heavy chain V-A2 region BS-1 is unknown. Furthermore, these protein sequences found in the membrane might be used to develop a vaccine against this pathogen. The 3D structures of these four proteins are obtained from proteins data bank (PDB) as shown in Figure 4.

## 4. Conclusions

A rise in drug resistance warrants the use of in silico approaches to identify targets unlikely to be similar to human proteomes. Using subtractive genomics approach, we identified novel protein sequences in *B. pertussis* strain B1917 that may be used for drug targets and novel compounds designed to target them. Additionally, four protein sequences are found in the membrane, which may help develop a *Bordetella pertussis* vaccine.

## Figures and Tables

**Table 1:** The various steps of subtractive genomics and their results.

S. No.	Steps	*B. pertussis*
1	Complete set of proteins	3550
2	Extracted paralogous (>80% identity) in CD-HIT	3085
3	No. of proteins non-similar to host using BLASTp (E-value 10^−3^)	2025
4	Vital protein sequences in DEG (E-value 10^−5^)	708
5	No. of vital membrane proteins (PSORT)	221
6	No. hypothetical protein as vital proteins (InterProScan)	19
7	Vital proteins involved in metabolic pathways (KEGG)	354
8	Essential drug target proteins (DBD)	08

**Table 2:** Non-similar vital proteins of *B. pertussis* strain B1917 similar to FDA-approved drugs against database of DrugBank using BLAST P.

Protein ID	Protein Name	Drug Bank ID	Drug Bank Organism
P00525	src-p60 phosphoprotein	DB08901 DB01268	*Rous sarcoma virus*
A0A385	Neuraminidase	DB00558 DB06614	*Influenza A virus*
P00526	src-p60 phosphoprotein	DB00171 DB00619 DB06616 DB04868	*Rous sarcoma virus*
P01827	Ig heavy chain V-A2 region	DB00098	*Oryctolagus cuniculus*
P01920	*MHCHLA-DC3-beta*	DB00254	*Homo sapiens*
Q31259	MHC H2-IA-alpha	DB00071	*Mus musculus*
P01920	*MHC HLA-DC3-beta*	DB00071	*Homo sapiens*
P25020	*src-p60 phosphoprotein*	DB05294	*Rous sarcoma virus*

**Table 3:** Non-homologue essential proteins having less similarities with the gut flora.

S. No.	Non-Homologue Essential and Non-Gut Flora Proteins	Virulence
1	Neuraminidase	Yes
2	Ig heavy chain V-A2 region BS-1	Yes
3	*MHC HLA-DC3-beta*	Yes
4	MHC H2-IA-alpha	Yes

## Data Availability

Not applicable.

## Abbreviations

NCBI	National Center for Biotechnology Information
CD-HIT	Cluster Database at High Identity with Tolerance
BLAST	Basic Local Alignment Search Tool
DEG	Database of Essential Genes
KEGG	Kyoto Encyclopedia of Genes and Genomes
FDA	Food and Drug Administration
